# Examining the critical factors of internal audit effectiveness from internal auditors’ perspective: Moderating role of extrinsic rewards

**DOI:** 10.1016/j.heliyon.2023.e20497

**Published:** 2023-09-30

**Authors:** Hamza Alqudah, Noor Afza Amran, Haslinda Hassan, Abdalwali Lutfi, Noha Alessa, Mahmaod alrawad, Mohammed Amin Almaiah

**Affiliations:** a Accounting Department, Faculty of Administrative and Financial Sciences, Irbid National University, Irbid, 2600, Jordan; b Tunku Puteri Intan Safinaz School of Accountancy (TISSA), University Utara Malaysia (UUM), Sintok, Kedah, 06010, Malaysia; c Department of Accounting, College of Business, King Faisal University, Al-Ahsa, 31982, Saudi Arabia; d Department of Accounting, College of Business and Administration, Princess Nourah bint Abdulrahman University, P.O. Box 84428, Riyadh, 11671, Saudi Arabia; e College of Business Administration and Economics, Al-Hussein Bin Talal University, Ma'an, 71111, Jordan; f Department of Computer Science, Aqaba University of Technology, Aqaba, Jordan; g King Abdullah the II IT School, The University of Jordan, Amman, 11942, Jordan; h Applied Science Research Center, Applied Science Private University, Amman 11931, Jordan

**Keywords:** Internal auditors' effectiveness, Empowerments, Extrinsic rewards, Public sector, Jordan

## Abstract

An effective internal auditor can support an organization in achieving its goals and protect its assets and funds. However, to be effective, the internal auditors need to be empowered with relevant resources. This study aims at probing the influence of the empowerments (management support, external auditors' collaboration, independence, size of internal audit units, and extrinsic rewards) on the internal auditors' effectiveness, and to examin whether extrinsic rewards moderate the association between respective empowerments and the effectiveness of Jordanian public sector internal auditors'. The current work relied on 117 surveys collected from Jordan's internal audit and financial managers in the public sectors. According to the Resource-Based View (RBV), the findings show that management support, external auditors cooperation, independence, and extrinsic rewards all have a significant influence on the effectiveness of internal auditor. The of the Internal Audit Department (IAD) size was discovered to be insignificant. Also, the results have revealed partial support for the influence of the extrinsic reward as a moderator. Because of the prominence of the public sector in the Jordanian market, this current research expands on the idea of enabling internal auditor (IA) to accomplish their assigned objectives. The findings might help authorities develop new norms and legislation for the internal audit profession. These empowering characteristics may also improve internal auditors' capacity to execute their duty in saving public monies and limiting corrupt practices inside public sector organizations.

## 1 Introduction

Internal Audit (IA) has undergone significant developments globally. Initially, IA's role was limited to verifying accounting records only. However, the focus later shifted towards monitoring financial compliance and Internal Control (IC), as highlighted by Kagermann et al. [1]. Despite these changes, the internal auditors' responsibilities have continued to evolve under the role of numerous factors [2,3]. Consequently, the Institute of Internal Auditors (IIA) updated IA's definition in 2020 to reflect these transformations as following:

*“Internal auditing is an independent, objective assurance, and consulting activity designed to add value and improve an organization’s operations. It helps an organization accomplish its objectives by bringing a systematic, disciplined approach to evaluate and improve the effectiveness of risk management, control, and governance processes”* (p.2).

The aforementioned description means that IAs have been adjusted to enhance and add consequence to the operations of an organization by offering a broad range of services, including the operational consulting and audit services.

The role of IAs has undergone a transformation with a greater emphasis on its effectiveness, as studies by Alqudah et al. [4] indicate that it can add value to organizations. Effectiveness of IAs refers to the of internal auditor ability to attain organizational goals, as discussed in studies by Alqudah [5] and Badara and Saidin [6]. When IA is successfully operated, implemented, and managed, it can help organizations achieve their goals and protect their assets by providing assurance regarding information security anti "Cybercrime" [[7], [8], [9]], as well as improving risks management, control effectiveness, and governance processe [10,11]. Moreover, various studies including Alqudah et al. [12], Assakaf, Samsudin and Othman [13], and Abuazza, Mihret, James and Best [14] suggest that an efficient IAs can play an essential part in supporting administration in detecting irregularities. Consequently, the internal auditors' tasks and responsibilities have increased, primarily within public sector bodies, owing to the transformation of their role.

The public sector IAs need more support and empowerments from the relevant stakeholders to be capable of doing their responsibilities effectively [15], whereby public sector's goals are usually wide, complex, and difficult to measure as compared to the private one [16,17]. The public sector serves a large segment of the society members while at the same time affecting their living standard [18]. The public fund and asset, therefore, can be protected, and the management of institutions needs to be strengthened by incorporating important functions [3,13,19]. Consequently, in the public sector, the importance of IAs has increased to reduce the risk of corruption. In this regards, Alzebam [20], p:[16], noted that “IA function in the government environment has been expanded considerably, in a bid to meet the demands for raising the transparency, integrity, and improved level of government service delivery”. Besides, According to research by Coram et al. [21], organizations lacking an effective IA function are more susceptible to corruption compared to those that have one. Hence, for IA to be beneficial to an organization, it must be efficient, as noted by Erasmus et al. [22]. Despite the crucial role IA plays in achieving objectives in public institutions, IAs cant execute their duties effectively without the needed authority. In this regard, the "International Standards for the Professional Practices of Internal Auditing” (ISPPIA), "2030 –Resource Management Standard" [23, p:11] states “the chief audit executive must ensure that IA resources are appropriate, sufficient, and effectively deployed to achieve the approved plan.” This implies that the IAs cannot complete their assigned objectives without relevant resources (empowerments) [23].

Prior studies have revealed that the public sector IA with limited empowerment cannot achieve its effectiveness [4,17,20,24,25]. For instance, in the Libyan public sector, Abuazza, Mihret, James and Best [14] revealed that the effectiveness of IAs is inadequate as a result of a lower organizational status of IAs department, constrained scope of IA's work, restricted usage of IAs staff career, and weakness cooperation between IAs and exterior auditors. Meanwhile, Alzeban and Gwilliam [20] conducted a study on the drivers that influence the effectiveness of IAs in the Saudia Arabia public sector. Their research revealed that IA department size, collaboration with exterior auditor, independence, and top management support significantly promote and influencing the empowerment of internal auditors' effectiveness. According to empirical studies carried out in Jordan, it has been argued that the IAs have not fulfilled their responsibilities and missions adequately. The effectiveness of IA is influenced by several antecedents such as the inadequate qualifications of internal auditors, insufficient independence, inadequate IA staff, the limited upper management support, and lack of the external auditors' cooperation [[26], [27], [28], [29]]. According to Alqudah et al. [5], these factors undermine the effectiveness of AI as a powerful instrument for combating corruption as well as enhancing the operations productivity among public sectors.

Based on the previous literature associated to the public sector IAs effectiveness, Jordan context of public sector IA, and ISPPIA delivered by IIA, this current work aims at investegating the impact of the empowerments (i.e., the external audit cooperation, the independence, the upper management support, the IA department size, and extrinsic reward) on the efficacy of internal auditor. Meanwhile, given the contradicting results of previous studies concerning respective empowerments, the present study also nominates extrinsic rewards as a setting within which the IA profession pass off that could provide a varied aspect of the present discussions in the IA. That to say, the different level of extrinsic rewards provided among public sector institutions may affect the relationship between those empowerments and the IA's effectiveness [30]. However, reviewing prior audit research revealed that extrinsic rewards have received slight attentions in IA research.

Further, the scant literature in public sector IA effectiveness [17,31] is one of the reasons to do this research, particularly in Jordan. Hence, there is a necessity to do more studies in such a context [32]. For instance, Madawaki et al. [33] declared that only a few empirical researches have been carried on IA. Also, Endaya and Hanefah [34] have confirmed on the imperative to do more studies to investigate the drivers influencing the IA effectiveness. Besides, to the best information of the researcher, no empirical work has integrated the extrinsic rewards as a moderator, in order to recognize their influence on the association between empowerments and the IAs effectiveness, especially in the context of public sector context. Further, Endaya and Hanefah [35] confirmed that there are inadequate theories utilized to promote the research on the successful of IA functions. Hence, the present study used the RBV by introducing the empowerments of the IA effectiveness as relevant resources to the internal auditors to enable them delivering their services competently.

As for the extrinsic rewards moderator factor, this variable was pioneer tested in the IA topic. This factor will advance the body of knowledge of IA by giving some awareness into the public sector IA function in Jordanian context and another Arab nations that share in the developmental and cultural issues. In addition, the present study proposes recommendations to overcome the deficiencies of the IA profession. Policymakers will be given a new perceptiveness associated with the empowerments that will probably influence the effectiveness of IAs’. these perceptions may serve decisions makers such as: Parliament, the Senate, and Cabinet to enhance the successful utilization of the IAs functions in Jordan.

The present study reviews the prior studies to provide related hypotheses. Further, it discusses the methodology utilized and presents the measurement of the variables. Next, it displays the analysis outcome. Finally, the discussion and conclusions are presented.

## 2 Literature review

### 2.1 IA effectiveness

An active IAs can sustenance an organization in reaching its purposes through several roles. For example, the role of IA is to evaluate adherence to procedures, plans, policies, and regulations, assess the risk management effectiveness and IC system, and scrutinize measures taken to protect assets [11]. In this regard, an organization that operates without satisfactory IAD bears much of difficulty in implementation of its activities [36]. Therefore, Unegbu and Kida [37] summarize that effectiveness of IA is greatly beneficial since it can empower the public associations to advance in their regular deeds.

Moreover, it was reported that the effectiveness of the IAs is influenced by the self-valuation appointed by the managers. The internal auditors success can be only estimated by the related stakeholders' presumptions [38]. In this regard, one of the early efforts is that Alzeban and Gwlliam [20] provided a survey to obtain feedback from each organization's IA unit. Based on the responses that managers gave, their study has investigated the effectiveness of the IA task. In this research, on one hand, chose financial managers (as one of the auditee) to evaluate the IA effectiveness in a semi-external way, and on another hand, chose the IA managers to get feedback on the effect of the upper management, exterior auditors' support, independence, IAD size, and extrinsic reward that are regarded as empowerments on the IA effectiveness by their perspective. By considering the RBV, the empowerments are internal factors within organizations that could affect IA effectiveness. Hence, the utilization of this theory in studying the IA effectiveness assumes to provide a great enhancement to the domain of IA.

### 2.2 Internal audit scenario in Jordan

Regarding the Jordanian Prime Minister (JPM) proclamation [39] Section (31), all Jordanian public sector units are obliged to employ an IA function. In order to maintain the pace of IA with changes and developments in the public sector, the proclamation has received various modifications, like No. (3) 2011 and No. (11) for 2015. Concerning the proclamation No. (3) for 2011, the public sector Standards of IA have been determined in Jordanian context. The article (8) of the proclamation defined the IA profession's planned purposes that must be addressed to ensure that: (i) all financial transactions are properly recorded and categorized in the financial statements, (ii) all instructions of IC and, technical and administrative performance, financial policies, and regulations are properly met, (iii) all obtainable human and financial resources are assigned based on the strategies and established regulations, and (iv) expressing the views on any technical, financial, or managerial subject upon the senior management's request [39]. In addition, the JPM announced guidelines that govern the public sector IA profession. Hence, to reach the planned goals of the Jordanian public sector IA, several regulations have been set to guide the IA [39]. The proclamation declared that IA must be linked straight to the Minister. It also emphasized the unconditional upper management support to the IA units and the effective cooperation by all the management levels. Further, the Edict assures the fulfilment of the recommendations offered by the auditors [39].

However, Jordanian media outlets have extensively reported on financial and administrative corruptions in public institutions, as per the annual reports of the Jordanian Audit Bureau (JAB) [40], the number of corruption cases related to the public money until the end of 2015 was 13,206 cases, reaching approximately $2.5 billion. Also, the annual reports of Integriity and Anti-Coruption Comision, covering a period from 2010 to 2016, highlighted that there were fluctuations in the number of corruption cases, amounting to 2122 cases. These cases, along with the increasing public debt, have led to the misuse of public funds, resulting in a decline in the services quality offered to people and a reduction in their standard of living [[41], [42], [43]]. Consequently, the results indicate that IA has not been effective in combating corruption and managing public resources in Jordan's public sector, despite the existence of clear objectives and guidelines governing its function. The IA has been unable to meet its goals of safeguarding and handling public fund. Hence, it is essential to conduct empirical work to determine whether enhancing IA's capabilities or vice versa could improve its effectiveness.

## 3 Underpinning theory and hypothesis development

### 3.1 Resource-Based View theory

The present study uses the RBV theory by including the empowerments to enhance the effectiveness of the IA as scarce resources for the internal auditor to perform his duties effectively. The RBV theory assumes that organizations are unique and have different capabilities and resources, according to Barney [44]. These resources can be tangible or intangible and are essential for an organization's success. Ahmad [3] notes that the RBV theory focuses on how an organization's resources affect its behavior and performance. Based on this, organizations strive to find distinctive attributes that can enhance their performance. Grant [45] further confirms that the RBV theory emphasizes the effect of internal, firm-specific factors on the performance and views organizations as collections of resources that combine to form organizational capabilities. Brysen Ackerman and Eden [46] refer resource as any asset that might be exploited to support an businesses to undertake its goals and execute effectively. In this vein, according to the RBV, the firm competitive advantage is based on a wide range of rare, inimitable and non-substitutable resources [44]. In the context of the present study, it can be said that the implication of internal organization resources is a key part of the sources of competitive advantage and organizational performance [44,45,[47], [48], [49]]. When organizations have plenty of relevant inner capabilities, they may execute strategies that support their efficiency [45,50,51].

Given the preceding discourse, it can be inferred that the RBV theory is a sound and credible concept which provides a logical and convincing account of how internal resources contribute to the development of effective strategies, consequently leading to favorable performance in terms of intellectual assets [3,[51], [52], [53]]. The RBV theory focuses on the benefits of the inner resources of the organization as the key source of the organization's performance and behaviour is extremely relevant in investigating the influence of the empowerments to the internal auditors' effectiveness [44,45,54]. Accordingly, the RBV theory confirms the internal resources of organizations in achieving an additional feature [55]. The present work aims to investigate the impact of organizational inner factors on the effectiveness of the IA function. Specifically, it seeks to identify the unique internal resources or empowerments that enable IAs to perform their tasks in an effective manner. This present research findings will provide insight into the factors influencing IAs effectiveness [3].

Therefore, the RBV theory is used in the present study in order to explain the impact of the independent variable (empowerments) on the IA staff effectiveness within the public sector of Jordan. Based on the RBV theory, the empowerments are components within organizations that affect the IA's effectiveness. The present work research model illustrates the effect of empowerments (as independent variable) on the internal auditors' effectiveness (as a dependent variable) (see Fig. 1). The support from the upper management to the internal auditor, the level of external auditors' cooperation, the size of IAD, the independency level of internal auditor, and the volume of extrinsic rewards are empowerments to enhance the internal auditors' effectiveness.

**Fig. 1:**
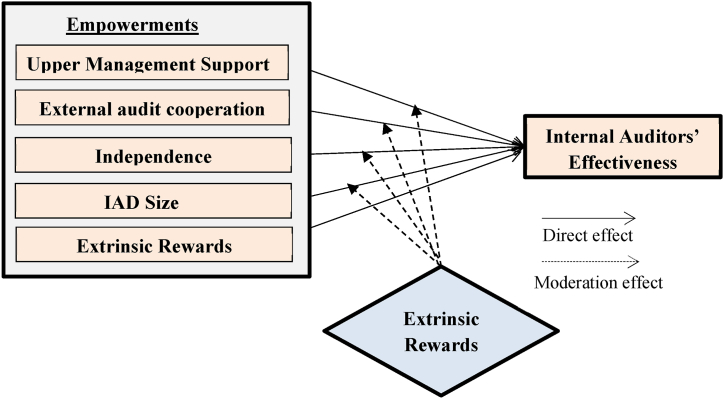
Research model.

### 3.2 Agency theory

Based on agency theory, an organization is perceived as an intricate web of contractual relationships between managers (agents) entrusted with the management of economic resources and the owners (principals). As a result of this theoretical paradigm, information asymmetry becomes pronounced due to agents' superior access to information over principals, impeding the latter's ability to rigorously determine agents' loyalty to principal interests. Inherently, the theory assumes that both agents and principals use contracts to increase their wealth. Corollary to this postulation is the emergence of the 'moral hazard' dilemma [56] characterized by agents taking actions detrimental to principal interests in order to advance their own interests [57][58]. During agent decision-making processes, principals are confronted with a paucity of information, making it difficult to discern the motives of agents. Structured contracts with control mechanisms, including the establishment of an Internal Audit Function (IAF), are envisaged as a means to alleviate this informational disadvantage, referred to as adverse selection, and simultaneously address the moral hazard problem.

As part of this agency paradigm, the public sector operates as well. Here, officials represent principals, advocating public interests, while agents are responsible for judicious resource management and aligning with public objectives. In order to mitigate inherent vicissitudes within this agency relationship, a robust IA initiative is essential [58]. Audits allow principals to assess the fidelity with which resources reflect their aspirations and to evaluate the financial representations of agents. Therefore, an external entity becomes pivotal to verify compliance, verify operational outcomes, verify the accuracy of financial reports, and scrutinize other relevant metrics.

IAF's inception within the Jordanian bureaucracy requires certain bonding expenditures on the part of public institutions (agents), in order to align with the accountability imperatives of citizens (principals). It is in this dynamic that the legislative body, which embodies public interests, assumes the role of the principal. As with conventional organizational expenses, these IAF-related expenditures are absorbed by the state exchequer to protect their diverse interests. In essence, the government capitalizes on the IAF to demonstrate its control over administrative apparatuses. Furthermore, a robust internal control framework is being pursued concurrently. IAF plays a crucial role in bridging information chasms between agent and principal in this tapestry.

The IAF must have adequate resources and institutional backing in order to fulfill its mandate effectively. Welbourne and Mejia [59] argue that performance-based incentives encourage a culture of mutual oversight among team members, which mitigates moral hazard. In light of the above, the present study utilizes the agency theory to identify the contingent impact of extrinsic rewards on the nexus between determinant variables and the efficacy of IAs. Therefore, we believe that calibrated extrinsic rewards for internal auditors can enhance the impact of empowerment variables on IA proficiency, particularly within the Jordanian bureaucracy.

### 3.3 Empowerments to the IA effectiveness

The factors that affect the internal auditor's effectiveness have been an argumentative issue. In this context, diverse factors have been directed by various academic research to examine IA effectiveness [22,60,61]. For instance, Ahmad et al. [62] observed that understaffed departments, bounded resources, the negative view of the IA role by the auditees, the deficiency of the internal auditors' proficiency, independence, and the restricted upper management support negatively influence the IA effectiveness. Likewise, Salehe [61] confirmed that lack of independence, competency, upper management support, external auditors' cooperation, and IAD size are vital factors that drive the insufficiency of the IA effectiveness. Furthermore, empirical research conducted within the Jordanian context consistently identified a lack of extrinsic rewards as an important impediment to internal auditor performance in the public sector [27,29,63,64].

In addition, the IAs are commonly criticized and blamed when they fail to detect corruption cases or misuse the public resources, but the internal auditors are not asked about the reasons behind their failure. Therefore, the present study attempts to highlight the factors that empower the effectiveness of the internal auditor in the public sector. Since the internal auditors without sufficient empowerments cannot conduct their works effectively, the present study aims at addressing the relevant empowerments to the Jordanian public sector internal auditors’ effectiveness. Among these determinants are the endorsement of upper management, collaboration with external auditors, the independence of auditors, and the size of the Internal Audit Department (IAD).

#### 3.3.1 Upper management support

The support of top management is one of the key drivers of IA effectiveness. The sufficient support by the senior management, will provide the IAD with sufficient resources to do its liabilities, obtain competint staff, present advancement, and continuous training [4,65,66]. In addition, ISPPIA confirmed the significance of the Chief Audit Executives (CAE) telling the highest management when there are any constraints in the budgetary of IA that hinder their performance [67]. So, such assist empowers the IAs units to achieve the appointed objectives within the organizations, thereby adding value to the organization by improving the organizational performance and processes [68,69]. The role of IA is crucial in assessing, scrutinizing, and ensuring the effectiveness of organizational activities beyond the reach of senior management. These levels are distant and inaccessible to them [70,71]. Hence, the IAs require good support and attention from the upper management to be able to perform the audit aims [4,5,25,72].

However, the increasing complexity and broadened scope of the public sector necessitate the indispensable support of upper managers for the success of IAD. Previous studies have confirmed that management's support is a critical factor in empowering the IA function and enhancing its effectiveness within this context [61,62,73]. For example, in a case study related to the IAs effectiveness services in the Ethiopian organizations by Mihret & Yismew [74], they revealed that the manager support strongly influences the IA effectiveness in the public sector. In their empirical study of the determinants influencing the effectiveness of Internal Audit (IA) within the Saudi public sector, Alzeban and Gwilliam [20] made a similar observation. As outlined in studies [22,34,75], this conclusion is consistent with other scholarly outcomes.

However, in the context of Jordan, there is a limited body of research on the effectiveness of management support for information architecture. However, the available literature suggests a positive link between these two variables, either explicitly or implicitly. For instance, two studies conducted on the IA units in the Jordanian ministries were by Rahahleh [64] and Ghneimat and Seyam [63]. Their studies indicated that the absence of upper management support was a vital obstacle that affects the effectiveness of IA. Therefore, the endorsement of upper management may play a pivotal role in determining the effectiveness of Internal Auditors (IAs). In addition, the Jordanian academic landscape presents a scarcity of studies examining the impact of upper management support on the effectiveness of information assurance.The objective of the current research is to investigate how the internal auditors' effectiveness in public sector entities is influenced by support from upper management, as perceived by the internal auditors themselves. The hypothesis of this study posits that providing adequate support to internal auditors from upper management will result in a greater degree of effectiveness in their work. Consequently, the following hypothesis is articulated:

H1 *upper management support have a positive affects on the public sector internal auditors' effectiveness.*

#### 3.3.2 External auditors’ cooperation

The external auditor's cooperation is an important part of the empowerments to IA effectiveness. A cooperative relationship between external and internal auditors refers to an integrative nexus that is characterized by unhindered access to working documents, a mutual exchange of insights, data, and analytical reports, all of which are intended to improve the quality of audit activities [20,76].Such cooperation not only facilitates efficiency in the audit processes but also adds value to the organization. Abu-Azza et al. [14] highlights the significance of this collaboration as it enables internal auditors to obtain vital information for evaluating risk control. The significance of internal and external auditors cooperation has been emphasized in professional standards due to its significant impact. The Coordination Standard of ISPPIA suggests that sharing information and coordinating activities is the minimum requirement for such cooperation (IIA) [23, p:10]. To establish a professional working relationship between these parties, it is essential to prioritize this cooperation. This can help internal auditors achieve their objectives and provide better services to organizations. Therefore, when considering the effectiveness of IA issues, the importance of such cooperation should not be overlooked.

According to Prawitt et al. [77], the partnership between internal and external auditors has led to a decrease in earnings management and an improvement in financial reporting quality. Therefore, it is recommended that public sector organizations cultivate a robust collaborative dynamic between their internal and external auditing contingents in order to maximize their alignment [[[78], [79]]. In light of this, it is imperative to provide comprehensive capacity-building and empowerment to internal auditors in public sector establishments, so that they are equipped with the skills, knowledge, and methodologies necessary to navigate the intricacies of their operational landscapes in an effective manner.Furthermore, Collaboration between effective external auditors has been confirmed by multiple studies to lead to increased effectiveness of IA activities [61]. Thus, proper external auditors' collaboration will increase the efficiency of IA, in that way support the public management in affording a good quality service [68]. IA effectiveness is often reduced by the lack of cooperation from external auditors, as noted in Ref. [75]. Despite the importance of the public sector in economic and social development, only a few number of works have examined the role of external auditors' cooperation and IA effectiveness in this sector, particularly in Jordan. Hence, this study predicts that a good cooperation between the public sector's internal auditors and the external auditors will produce the effectiveness of IA. This drives to the next hypothesis:

H2 *The external auditors' cooperation has a significant influence on public sector's IAs' effectiveness.*

#### 3.3.3 IAs’ independence

The internal auditors' independency has been known as the foundation of the IA definition and critical empowerment to the IA effectiveness [4]. This definition is derived by the IIA which stated that: "the internal audit activity must be independent, and internal auditors must be objective in performing their work" (IIA) [23, p:3]. In addition, according to the professional standards followed globally, adequate independency can be attained by reporting to levels within an organization that empower the IAD to perform their duties without interference and grant them authorized access to employees, records, and departments, avoid the conflict of interests, have a straight link with the upper management or board, and have the power to appoint and remove the CAE not by the control of the executive management, and not conducting a non-audit work [20]. Thus, if the IA staff are autonomous, that will increase the integrity of their works and reports [80,81] and accountability. According to Vanasco's [82], the attainment of the IA's desired outcomes is only possible if it operates independently. Therefore, the organizational unit responsible for IA must have a significant level of autonomy to execute its functions effectively. Consequently, the effectiveness of the IA is enhanced by the empowerment provided by the independence of internal auditors, since independence probably decreases the conflict level between the loyalty to the employer and loyalty to the specific managers, as well as provides the auditors with a suitable work condition that could empower them to do their task freely [14].

Accordingly, the internal auditors should be independent within an organization to obtain a high level of IA effectiveness [4]. For instance, Salehi [61] confirmed that the independence of IAD is the main empowerment of the effectiveness of IA in the Iranian listed companies. Besides, Numerous studies have established that the autonomy of IA is a vital factor in enhancing the effectiveness of IA in the public sector. This finding has been supported by multiple studies, such as Khelil, Akrout, Hussainey and Noubbigh [83], Poltak, Sudarma and Purwanti [84], Coetzee and Erasmus, Pududu, Malan and Legodi [85], Erasmus and Coetzee [22], and Usang and Salim [86]. Evidence from research conducted in Jordan also indicates that the absence of IA's independence is a major hurdle that hinders its effectiveness. In this context, further studies are necessary to be carried out in the developing countries, especially in Jordan due to lack of studies in the public sector. Concurrently, most previous studies have focused on CAE in this relationship context, whereas The impact of IAs' independence on the effectiveness of IA, as perceived by IA managers, has been overlooked in Jordanian context. Thus, the current research aims to examine how internal auditors' independence serves as an empowerment factor for improving the efficiency of IA, while considering the views of IA managers.

Therefore, this study predicts that the independency will empower the effectiveness of IA's. The aforementioned arguements lead to the third hypothesis:

H3 *The IAs' independence has a positive effect on the public sector internal auditors' effectiveness*.

#### 3.3.4 Internal Audit Department size

To effectively carry out its duties and responsibilities, the IA function requires adequate resources. Alzeban and Gwilleam [20] emphasize the importance of empowering IA with sufficient resources. The "Resource Management Standard" [87], p:11] outlined by ISPPIA further emphasizes that the CAE must ensure the availability of adequate resources to execute the appropriate plan. The term 'adequate' implies that the IAD should have a suitable size to discharge its responsibilities competently, as highlighted by Alzeban and Gwilleam [20]. Due to the complexity of the IA process, multiple disciplines of skills and knowledge are necessary to provide valuable insights into the company's business [88]. A larger IAD is expected to have a hierarchical administrative structure, with managers having separate control over the IAs teams [12,89]. Conversely, in smaller IADs, auditors may be responsible for more extensive tasks and engage in several types of engagement [90]. Therefore, when there is sufficient staff in the IAD, the effectiveness will probably increase [61,81,91,92]. This usually happens within the public sector institutions that have been recognized with additional complexity and risks, as well as many corruption cases [93,94].

In the Malaysian context, Ahmd et al. [62] confirmed that an inadequate number of internal auditors can be as a key issue that limits the Malaysian public sector's IA effectiveness. Besides, according to Dascalu's [95] findings and Rogala and Wereda's [91], the lack of a properly defined size for the IAD poses a challenge for IA in the public sector. Anderson et al.'s study in Ref. [96] supported this notion, stating that determining the appropriate IAD size depends on the particular tasks that IA performs, and should be adjusted according to factors such as the technology used, the expertise of IA staff, and the geographical areas involved.

Prior studies related to the IAD size and the public sector have gained very barely attention. Thus, more studies are essential in developing countries, including Jordan. The present study addresses the influence of the number of IAs as a proxy for IAD size on the IA's effectiveness. Specifically, the present study predicts that IAD within the public sector with a sufficient number of IA staff will empower the IA's effectiveness. Thus, we proposes the following hypothesis:

H4 *IAD size has a positive effectson the public sector internal auditors' effectiveness.*

#### 3.3.5 Moderating effect of extrinsic rewards

Portar and Lawlar [97] proposed two categories of rewards: intrinsic and extrinsic. Intrinsic rewards are directly tied to the job content itself, meaning that individuals receive them as a result of effectively accomplishing their tasks [4]. On the other hand, extrinsic rewards originate from external factors such as promotional opportunity, working condition, pay satisfaction, and advantages, which are provided by organizations with the aim of motivating performance [98,99]. However, extrinsic reward is more strongly correlated with employee commitment than intrinsic, as organizations have more direct control over extrinsic rewards. As a result, extrinsic rewards are vital to inspire employees, such as IAs, to exhibit creative behaviour [98].

Pay satisfaction is an extrinsic reward that indicates the satisfaction with the paid value obtained from the magnitude of work performed, as well as satisfaction with pay parallel to the value paid in similar organizations. Pay satisfaction has been observed to significantly influencing job attitudes. Hence, according to Shahnawaz and Jafri [100], organizations with satisfied staff tend to operate more efficiently than those without. Heneman et al. [101] further support this notion by stating that pay satisfaction is a crucial aspect of measuring organizational effectiveness. Nevertheless, there is inadequate research on how the internal auditor's pay satisfaction affects IA performance.

According to Rust et al. [102] the working conditions provided by an organization can impact the job attitudes of its employees, including internal auditors. As internal auditors dedicate a significant portion of their day to work, a favorable working environment can serve as a rewarding factor and foster loyalty towards their employer [98,103]. Research suggests that providing good working conditions is an essential extrinsic rewards for internal auditor to complete their tasks in effective manner in an organization.

Fringe benefits refer to external incentives that reflect an employee's contentment with the supplementary advantages offered by a company on top of their usual salary. This encompasses not just what the organization provides but also how it measures up against the offerings of other companies [99]. These benefits are considered essential aspects of human resources practice applied by companies to maintain a devoted and content workforces. Examples of common fringe benefit include medical insurances, housing allowances, use of business vehicles, meals, and sick pay [104].

Finally, according to Malhotra et al. [99], employees' satisfaction and perception of adequacy with regards to an organization's promotion policy are what define promotional opportunities. These opportunities for career growth and development play a critical role in human resource management practices aimed at fostering organizational commitment among employees. Given this, Young et al. [105] argue that agreement on promotional opportunities stands out as one of the most significant determinants of organizational commitment.

Based on recent research [64,106], internal auditors in the public sector, much like any other employee, are incentivized by rewards for their efforts and tend to perform better when faced with challenging tasks. Offering monetery reward can incraese motivations and improve their productivity, which in turn enhances the overall effectiveness of IA. This is particularly important in Jordan, where poverty rates are high and wages are lower than average [107]. Therefore, providing attractive extrinsic reward could help encourage internal auditor to work harder and be more motivated in carrying out their duties effectively.

While prior research has explored the potential association between external reward and employee performance or obligation across various fields [99,108,109], there are a lack of works examining how extrinsic rewards can enhance internal auditors' effectiveness. Thus, this research seeks to explore the impact of extrinsic rewards on IA effectiveness within Jordan's public sector. Accordingly, the present study proposes that the IA staff awarded with high extrinsic rewards will lead to greater IA effectiveness. This drives to the following:

H5 *Extrinsic rewards positively affects the public sector internal auditors' effectiveness*.

Besides, Auditors commonly encounter difficulties when performing their tasks, which can stem from various factors inherent to the job [82,110], including insufficient resources, work pressures, and task uncertainties and complexities [111]. These challenges often result in inaccurate audits and a lack of consensus among auditors, ultimately impeding audit quality [112]. To address this issue, audit companies recognize the value of providing rewards to incentivize external auditors to perform their duties more effectively [113]. Similarly, internal auditors also require rewards to motivate them to conduct their tasks efficiently, particularly in complex environments like public institutions [99].

Howevere, from the prespactive of the Agency theory, for IAF to function effectively as an agents, it demands sufficient resources and provision to carry out its responsibilities competently. Whereby, Paying for teams performance results in teammates reciprocally monitoring each other, thereby reducing moral hazard [59]. Hence, the Agency theory has been employed in this work to substantiate the contingent impact of extrinsic rewards on the link between various factors and IA effectiveness. Therefore, extrinsic rewards can contribute as a significant moderating role in the relationship between addressed empowerments and the internal auditor's effectiveness, particularly in the public sector of Jordan. The reason behind the complexity of tasks for internal auditors can be attributed to a combination of factors such as high poverty rates, low wages, and elevated prices [107]. This is further compounded by the intricate workings of Jordanian public institutions, as highlighted by Rahahleh [28]. Hence, the present study claims that the strength of the relationship between addressed empowerments and the IA's effectiveness can be moderated by the internal auditors' perceptions of extrinsic rewards. According to this argument, it is rational to indicate that the level of extrinsic rewards may affect the internal auditor behaviour irrespective of whether the support of upper management is high, or there is good cooperation from external auditors, or whether there is an appropriate level of independence, or whether the IAD size is sufficient. Hence, the relationship between IA effectiveness and its empowerments may be moderated by extrinsic rewards. This drive to formulating the next hypotheses:

H6 *Extrinsic rewards moderate the effect of UMS on the IA's effectiveness.*

H7 *Extrinsic rewards moderate the influence of external audetors' coperation on the IA's Effectiveness.*

H8 *Extrinsic rewards moderate the influence of internal auditors' independence on their effectiveness.*

H9 *Extrinsic rewards moderate the influence of IAD size on the IA's effectiveness can be moderated by the extrinsic rewards.*

## 4 Research model

The present research has built upon the frameworks advanced by Alzeban and Gwalliam [20], given their pertinence to Jordanian legal provisions and their consonance with the International Standards for the Professional Practice of Internal Auditing (ISPPIA). Alzeban and Gwalliam's seminal work delineated five critical determinants, specifically: management endorsement, competencies of internal auditors, independence of internal auditors, the size of the Internal Audit Department (IAD), and collaboration between internal and external auditors, all influencing Internal Audit (IA) efficacy. Upon an intricate examination of the analytical unit corresponding to each determinant, it was discerned that, predominantly, these determinants operated at the organizational echelon. Notably, the competency of internal auditors was an exception, being at the individual stratum, and hence was precluded from the analysis.

Nonetheless, the extant research by Alzeban and Gwalliam [20] exhibits a notable lacuna: it neglects to explore the potential ramifications of extrinsic rewards on the Internal Audit (IA) effectiveness, particularly within the public sector milieu. To ameliorate this scholarly void, the current study incorporates several foundational frameworks: i) the International Standards for the Professional Practice of Internal Auditing (ISPPIA) as promulgated by the Institute of Internal Auditors (IIA), ii) antecedent academic investigations, and iii) relevant legislative statutes in the Jordanian public sector. Accordingly, extrinsic rewards are introduced as an additional, influential determinant that could potentially modulate IA effectiveness. These extrinsic rewards are analytically operationalized as a contingent variable, designed to mediate the links between the independent variables and their subsequent impact on IA effectiveness. Hence, the study posits that these independent variables serve as shaping factors in determining IA effectiveness, but their influence is further moderated by the presence of extrinsic rewards, as depicted in Fig. 1.

## 5 Research design

In this study, the financial manager and IA's manager in the public sector of Jordan that are under the oversight of JAB were the target participants. The total population was 287 government entities. Each government entity has received two hard copies of the questionnaire. One questionnaire was directed to financial managers to cover the IA's effectiveness, whereas the second questionnaire was directed to IA managers to address the independent variables. In this research, GPower tool was employed to ascertain the minimum necessary sample size. It comprised an error type (α) value of 0.05, an effect size of 0.3, a power of 0.95, and 5 predictors. The determined minimum required sample size was 111. In total, 117 valid questionnaires were utilized for subsequent analysis. Of the 287, 117 valid responses were received; 17 were from ministries, 6 were from public universities, 16 were from independent bodies, and 78 were from governorates. Wherein, 69 institutions had one (1) to 4 staff in their IAD, 35 had from five (5) to eight (8) internal auditors, and 13 had above nine (9) internal auditors. Ninety-one per cent (91%) of the IA managers were men. The average age was 40 years old, and their average work experiences were 10.5 years approximately.

Furthermore, the present study employed the financial managers' (as one of the auditee) perceptions as a ‘semi external’ measurement of the IA's effectiveness. IA effectiveness items were developed by Alzeban and Gwilliam [20], where the items were measuring the IA effectiveness based on the managers perception. On another hand, the IAs managers' responses have been utilized to evaluate the empowerments (independent constructs). Thus, the independent variables represent the IA managers' perceptions concerning empowerments to their effectiveness. A 5-point scale was used to measure all items (1 = strongly disagree to 5 = strongly agree). Appendix 1 presents the measurements of the study variables. Finally, prior to the data collection process, the final copy of the questionnaire was prepared. The informed consent was attached with the questionnaire. All respondents were asked to sign the consents before filling the questionnaire. In the consent we explained research aims and the main concepts for participants' understanding. All participants voluntarily participate in this questionnaire survey. In addition, we confirmed the privacy of respondents to improve the respondent sincerity and decrease the socially desired responses.

## 6 Data analysis

The current study used the Partial Least Squares Structural Equation Modeling (PLS3-SEM), for data analysis. As indicated in Appendix 1, the mean score for the latent factors were greater than the midpoint on the one to five scales, whereas the IAD size mean score was less than the middle-point, which infers that the IAD in the public sector of Jordan is insufficient in size considering its need. Regarding the effectiveness, the mean score of 3.067 suggests that the Jordanian public sector internal auditors meet the defined objectives confirming the compliance with policies, plans and regulations of the public entities.

The PLS-SEM was used to verify the hypotheses of the present study. PLS-SEM is recommended for the path modelling approach among practitioners and scientists [[114], [115], [116], [117]]. PLS provides a valuation of the associations between latent constructs with different items, even with a small sample size or with a complicated model. Also, it could be applied to model the hierarchical constructs at different abstraction levels [118]. PLS also works competently when the proposed model involves many structural path associations [[119], [120], [121]]. Furthermore, the rationale underpinning the adoption of the PLS-SEM (Partial Least Squares Structural Equation Modeling) approach is multifold. Firstly, the primary objective of this investigation is not merely to confirm or negate specific theoretical constructs, but rather to delve into and prognosticate the interrelations between the independent and dependent variables. Specifically, the emphasis is on elucidating the variance inherent in the effectiveness of Internal Audit (IA). Secondly, the PLS-SEM methodology, in its own right, possesses the analytical acumen to both scrutinize and validate theoretical frameworks. Thirdly, the research model that undergirds this study is characterized by reflective constructs and encapsulates an interaction effect. Given that the PLS-SEM framework offers a malleable architecture in its specification of both measurement and structural models, it becomes manifestly apt for modeling research paradigms of this nature, rendering it the most judicious choice for analytical exploration.

The PLS route model is made up of two basic models: the measurement model and the structural model. The measurement model investigates how constructs connect to their measures, whereas the structural model depicts the links between the constructs themselves [118]. The first step in starting the PLS analysis is to run validity and reliability tests on the measurement model. Hair et al. [118] suggest that during this stage, any indicators with loadings ranging from .40 to .70 should be eliminated from the scale if doing so results in an increase in either the Average Variance Extracted (AVE) or Composite Reliability (CR) above the recommended threshold value. As a result, all the items loading was more than 0.40. Besides, some items were kept (that range from 0.40 to 0.70). Hair et al. [116] recommended to increase the AVE and CR values of the related latent constructs to the lowest acceptable value, thereby requiring the removal of several indicators from these latent variables to build trustworthy reliability, namely, EAC1, EAC2, EAC4, EAC9, IAI2, IAI3, IAI4, IAI8, IAE14, IAE15, ER1, ER3, ER5, and ER9. Hence, after eliminating 14 indicators that were badly loaded, 35 indicators remained. Eliminating items is viewed as common and normal in some studies [12,118,122].

The findings of the CR and cross-loading indicators for Convergent Validity are shown in Table 1. (CV). The AVEs of all constructs above the.50 criterion [116], showing that each variable explains more than 50% of the variation in its own indicators, exhibiting significant CV. Furthermore, each latent construct's square root value of the AVE was bigger than its relationship with other latent constructs, suggesting a higher level of Discriminant Validity (DV). These results in Table 2 demonstrate that the square root of AVE were all bigger than the relationships between the components, indicating discriminant validity. The study's outputs indicate that all construct CR values surpassed the threshold of .70, which confirms the model's reliability [116,123].

**Table 1:** Convergent validity.

Constructs	Mean	Items name	loading	VIF	CR	α	AVE
Internal auditors' effectiveness (IAsE)	3.067	IAsE1	.726	2.009	.928	.916	.5
		IAsE10	.657	1.929			
		IAsE11	.703	2.086			
		IAsE12	.634	1.772			
		IAsE13	.628	1.679			
		IAE2	.706	2.041			
		IAsE3	.718	1.974			
		IAsE4	.758	2.965			
		IAsE5	.754	2.875			
		IAsE6	.739	2.391			
		IAsE7	.719	2.115			
		IAsE8	.756	2.114			
		IAsE9	.671	1.972			
Internal Auditors' Independence (IAsI)	3.153	IAsI 1	.637	1.301	.83	.746	.5
		IAsI 5	.651	1.267			
		IAsI 6	.731	1.252			
		IAsI 7	.756	1.917			
		IAsI 9	.732	1.548			
External Auditor Cooperation (EAsC)	3.134	EAsC 3	.626	1.244	.85	.777	.53
		EAsC 5	.713	1.423			
		EAsC 6	.75	1.702			
		EAsC 7	.767	1.711			
		EAsC 8	.777	1.595			
Upper Management Support (UMS)	3.134	UMS 1	.675	1.416	.87	.82	.53
		UMS 2	.721	1.563			
		UMS 3	.732	1.49			
		UMS 4	.763	1.712			
		UMS 5	.739	1.664			
		UMS 6	.717	1.538			
Internal Audit Department Size (IADS)	1.601	IADS	Single item construct				
Extrinsic Rewards (ERs) “Moderator”		ERs 2	.686	1.403	.84	.757	.51
		ERs 4	.736	1.515			
	3.088	ERs 6	.719	1.503			
		ERs 7	.711	1.329			
		ERs 8	.703	1.342

**Table 2:** Discriminant validity.

Constructs	EAsC	ERs	IAsDS	IAsE	IAsI	UMS
EAsC	.73					
ER	.51	.71				
IAsDS	.06	.02	1			
IAsE	.59	.62	.08	.71		
IAsI	.46	.55	−.07	.59	.71	
UMS	.41	.36	.05	.58	.32	.73

To further assess potential multicollinearity concerns, the Variance Inflation Factor (VIF) was calculated for each construct [117]. Hair et al. [116] defined non-problematic multicollinearity as a VIF value below five and a tolerance greater than 0.20. As shown in Table 3, all constructs registered VIF values below the prescribed limit, indicating the absence of collinearity complications. The empirical results demonstrate the robustness and reliability of the measurement model, thereby qualifying it for further analysis.

**Table 3:** Multicollinearity evaluations.

Variables	Tolerance	VIF
UMS	0.776	1.289
IAs’I	0.584	1.712
EAs’C	0.567	1.764
ERs	0.583	1.716
IA D	Single item construct	

Concerning the structural paths (model), since the model of this study contains moderator, the structural model should be at first executed without the (interaction effect) moderator [116]. Hence, two models have been used in the present study: direct and indirect effect model. Furthermore, for hypothesis testing, the present followed a one-tailed test [116], considering a t-values between 4.466 and 0.9993 to be significant at the level of 0.05, while the values exceeding 2.330 was deemed significant at the level of 0.01.

The findings of the direct effect model (standardized path coefficient and the significance value) were used for testing the hypotheses H1, H2, H3, H4, and H5. The path coefficients and their significance level for each latent construct in the structural model were measured utilizing the PLS algorithm and the PLS bootstrapping procedures utilizing a resample of 5,000, employing a one-tailed test [116]. Table 4 results displays that the influence of the upper management support on IA's effectiveness was significant and positive (β = 0.311; *p* < 0.01), thereby demonstrating that as the support of upper management increases, IAs' effectiveness will increase too. Hence, H1 was supported. Regarding the role of the external audit's cooperation on the IA's effectiveness, the result revealed a significant and positive effect (β = 0.21; *p* < 0.01). When the external audit's collaboration rises, the IA effectiveness will be increased too, which supports H2. Regarding H3, the result shows the significant positive association between the internal auditors' independence and the effectiveness of the IAs (β = 0.252, *p* < 0.01), thereby demonstrating that as the extent of the IAs' independence increases, IAs' effectiveness increases too. IAD size was, however, found to be insignificant (β = 061; *p* = 0.168) which represents that the IAD size has no influence on the internal auditors' effectiveness at the significance level 0.05. As a result, H4 is not supported. Finally, the association between the extrinsic rewards and the effectiveness of the IAs was significant and positive (β = 0.264, *p* < 0.01). This result reveals that as the extrinsic rewards increase, the IAs' effectiveness will increase as well, thereby supporting H5. As presented in Tables 4 and it was found that four factors have a significant influence on achieving the IAs' effectiveness, whereby upper management support has the strongest influence, while IAD size has no influence. The model of the present study explains 62% of the dependent variable which suggests a moderate exploratory power. Chin (2010) proposed a general guideline that R^2^ values of o.67, o.33, and o.19 can be considered substantial, moderate, and weak, respectively.

**Table 4:** Direct effect hypotheses assessment.

Hyp	Path coeffi	T- value	*p*-value	Result
H1: UMS - > IAE	.311	3.96	.000**	Supported
H2: EAC - > IAE	.210	3.27	.001**	Supported
H3: IAI - > IAE	.252	3.83	.000**	Supported
H4: IADS - > IAE	.061	.963	.168	Not supported
H5: ER - > IAE	.264	4.27	.000**	Supported

**Notes:**$R^{2}=.62$ percent.

*Note:* *p-values <0 0.01; **p-values <0 0.05. IAE – internal auditors' effectiveness; UMS – upper management support; EAC – external auditor cooperation; IAI – internal auditor independence; IADS – internal auditing department size; ER – extrinsic rewards.

To the moderator effect of extrinsic rewards, a model was implemented by offering four interaction (indirect) effects on the link between exogenious variables and IA effectiveness. Then the model was observed by applying 5000 procedures of bootstrapping. Table 5 presents the results of the indirect effect model for hypotheses H6, H7, H8, and H9. As the interaction effects were non-directional, a two-tailed test basis was utilized to evaluate the results, following the method outlined in Hair et al. [116]. The table displays the standardized path coefficients and their corresponding significance values. As shown in Table 5, the interaction between extrinsic rewards and upper management support on internal auditors' effectiveness is significant and positive (β = 0.13423, *T*-value = 2.0289, *p* < 0.05). Thus, extrinsic rewards have a positive contingent effect on the interrelation between upper management endorsement and IA effectiveness. There is a positive relationship between upper management backing and the effectiveness of the IA when there is an elevated level of extrinsic rewards. Therefore, Hypothesis 6 (H6) was supported by empirical evidence. Moreover, the t-value representing the indirect impact between extrinsic rewards and external auditors' collaboration regarding the effectiveness of IAs was statistically non-significant (β = 0.083, T-value = 1.042, p > 0.05).Thus, H7 was not supported. Besides, for the indirect effect between extrinsic rewards and independency on IA effectiveness, the T-value is insignificant (β = 0.075, *T*-value = 1.003, *p* > 0.05). Thus, H8 was rejected. For interaction effect between extrinsic rewards and IAD size on internal auditor's effectiveness is significant and positive (β = 0.203, *T*-value = 3.17, p < 0.01). Accordingly, extrinsic rewards have a positive moderating effect on the association between the size of the Internal Audit Department (IAD) and the effectiveness of internal auditors. A higher magnitude of extrinsic rewards augments the positive correlation between the dimension of the IAD and the effectiveness of internal auditors. Based on these findings, Hypothesis 9 (H9) is empirically validated.

**Table 5:** Interaction model Findings.

Hyp		Path coeffi	T-statistics	*p*-values	Decision
H6:	ER*UMS- > IAE	0.13423	2.0289	0.041	Supported
H7:	ER*EAC- > IAE	0.083	1.042	0.149	Not supported
H8:	ER*IAI- > IAE	0.075	1.003	0.158	Not supported
H9:	ER*IADS - > IAE	0.203	3.17	0.003	Supported

In addition, the incorporation of the moderating effect resulted in an increase in R^2^ from 0.62 to 0.628. An indication of the magnitude of the moderator's effect (f^2^) is provided by the differential in R^2^. Based on Cohen's benchmarks, effect sizes of 0.35, 0.15, and 0.02 are classified as large, moderate, and small, respectively [124]. To determine the effect size, Cohen's prescribed equation was used to calculate R^2^s for both the direct model and the interaction paradigm. In this way, the effect size attributable to the interaction model was calculated to be 0.0215, which is considered to be a small effect size. In this study, the interaction's f^2^ effect is observed to be modest.

## 7 Discussions and conclusions

The aim of this study was to investigate how empowerment affects the effectiveness of internal auditors, while also examining the role that extrinsic rewards play in this relationship. The RBV theory predicts that organizations require sufficient resources to achieve excellent results, and this study confirmed this prediction through its model. All of an organization's capabilities and assets can be considered resources that empower them to reach their goals, which in turn enhances the effectiveness of the organization. An internal auditor's effectiveness can be enhanced by a variety of internal resources, including upper management endorsement, autonomy, aspects of the IAD, synergies with external auditors, and extrinsic incentives. The findings of this research provide salient insights into the mechanisms by which internal auditors might be bolstered in order to achieve heightened effectiveness, emphasizing especially the moderating effect of extrinsic rewards, as posited by the Resource-Based View (RBV).

The data analysis were divided into two models (direct and indirect effects model). In the direct effect model, the results of the PLS path model showed that all the constructs significantly influence the IA effectiveness, except the IAD size. Specifically, the results showed that the upper management support, external auditor's cooperation, internal auditors' independence, and the extrinsic rewards have a significan influence on the IA's effectiveness. The significant association between the UMS and the IA's effectiveness is consistent with the forecast of the RBV [125,126], which suggests that sufficient internal resources are needed to attain effectiveness [127]. Thus, the internal auditor's effectiveness can be reached by adequate empowerments including the upper management support. When the internal auditors are empowered with sufficient support from the upper management (i.e., response to their reports, IAD has adequate budget), it will result in a great effectiveness level of IA. Therefore, the support from the upper management within the Jordanian public sector can be expected to empower the IA's function to protect public funds and resources. The upper managemtableent support can empower the internal auditors to combat corruption and adds value to the Jordanian public institutions. Consequently, this outcome is consistent with most of the related literature [20,61].

As for the external auditors' cooperation (H2), the findings show that the external auditor's cooperation has a positive influence on the internal auditor's effectiveness significantly. Wherein the high external auditor's collaboration level drives to enhancing the internal auditor's work. As a result, Hypothesis 2 (H2) is empirically supported. Observations such as these align with the concepts and view of the RBV theory [44], which asserts that human collaborations serve as intrinsic resources, which enable an organization to achieve its goals. Additionally, this outcome is consistent with antecedent research [4,84,86]. Ahmad's study [3] demonstrated the beneficial effect of external auditor collaboration on the IAs effectiveness in Malaysia's public sector. The coordination between external and internal auditors can enhance the competence of internal auditors, making management capable of delivering commendable public services as a result of their efforts. Consequently, the current work concluded that the external auditor's cooperation (Audit Bureau staff), by exchanging reports, ideas, and information, leads to non-duplication in the audit works and increases audits' quality, which in turn, leads to further IAs' effectiveness. Therefore, the present study confirmed that the external auditor's collaboration is an integral part of the empowerments to the effectiveness of IA in the public sector of Jordan.

Independence can facilitate internal auditors in executing their duties devoid of external constraints or pressures [22].In parallel with the RBV theory, if a public institution has unique internal abilities to a satisfactory extent, the institution will be able to improve its effectiveness [44]. So, this study hypothesized that the IAs' independence has a significant influence on their effectiveness significantly (H3). This study concluded that proper independency level will enhance the effectiveness of the IAs' tasks. The result is consistent with the RBV theory's expectation, and preceding studies that referred to a notable relationship between the internal auditor's independence and his effectiveness [61]. It is unavoidable for any institution in Jordan to not give the internal auditors free access to any department without the interference of the upper management, as well as does not engage them in works out of their responsibility.

According to the findings of the present work, the size of an IAD has a significant impact on the efficacy of internal auditors. The number of employees in the department defines the size of IAD, according to previous research [61,91]. This indicates that the number of employees in an IAD plays a critical role in enhancing the effectiveness of internal auditors, particularly in public sectors that face additional risks, complexities, and cases of corruption [93]. Additionally, various studies have established a positive correlation between the size of IAD and the effectiveness of IAs [61]. Therefore, the present study proposed that there exists a significant and positive association between the size of IAD and the effectiveness of internal auditors (H3). Contrary to expectations, the size of the IAD did not significantly impact the effectiveness of the IA in entities. This finding diverges from the RBV theory and prior research conducted by Alzeban and Gwilleam [20], Barniy [44], and Saleh [61]. One possible explanation for this outcome could be attributed to variations in the size and scope of services provided by the entities included in the study. Notably, some entities had fewer than three IA member, while others employed more than 13 or even 20 IA professionals within their IAD, leading to a substantial difference in IAD sizes across the surveyed public entities. The results of this study align with previous research conducted by Ho and Hutchinson [100], which found similar results regarding this specific relationship. Additionally, the size and functions of public sector institutions vary, leading to differences in the size of their IAD. The ISPPIA's "Resource Management Standard" [87, p:11] further emphasizes that the CAE must ensure that IA resources are sufficient to achieve the appropriate plan. With this regard, "adequate" refers to having a competent IAD size that considers entity size and structure. The embodiment public sector for different branched entities (e.g., ministries, governorates, independent bodies, public universities) has resulted in a large gap between the IAD size. The findings of the present study indicate that the majority of the Jordanian public entities have less than four staff in their IAD since most of the surveyed public entities were governorates, which are smaller in size compared to ministries that are bigger and have more complex objectives and tasks.

In addition to analyzing IA's effectiveness, this work also explored the impact of extrinsic rewards. These rewards are derived from external factors that are not related to job functions (such as benefits, paying satisfaction, working condition, and promotional opportunities), and are administered by organizations to motivate performance [99]. Research has shown that organizations with satisfied employees tend to be more efficient than those without [100], and therefore, extrinsic rewards play a crucial role in stimulating creativity among internal auditors [98]. Similarly to other employees in any organization, internal auditors in the public sector have a tendency to set goals for themselves based on the rewards they receive, which ultimately leads to greater level of performance [64]. The commitment of an organization to provide monetary reward has been shown to increase the motivation and productivity of internal auditors, leading to greater effectiveness in IA. This aligns with the prediction of the RBV theory, which views the organization as a combination of resources that contribute to its abilities. Bryson et al. [46] explain that these resources are assets that can be leveraged to help achieve organizational objectives and improve operations. Therefore, extrinsic rewards, such as those provided by an organization, can impact the performance of IAD as an internal factor. This study's findings suggest that extrinsic rewards play a significant role in enhancing the effectiveness of IA. Consistent with the RBV theory [46], the findings imply that public sector organizations which offer higher levels of extrinsic rewards tend to achieve greater effectiveness in internal auditing. Nonetheless, the Jordanian context is characterized by a significant proportion of poverty, low salaries, and high costs [107,128,129,130]. Hence, attractive extrinsic incentives might empower internal auditors to perform at their best and enhance their motivation towards achieving optimal outcomes.

The Agency theory has been employed in the present study to substantiate the moderating effect of extrinsic rewards on the relationship between various factors and IA effectiveness. The study focused on the indirect effect model and identified the variable of extrinsic rewards as a moderator in the relationship between various factors, such as UMS, external auditors' cooperation, independence, and IAD size, and the effectiveness of IAs in Jordan's public sector. The study formulated hypotheses H6, H7, H8, and H9, each associated with extrinsic rewards as a moderator. The results, presented in Table 5, indicated that two hypotheses (H6 and H9) were supported by the findings. In line with the agency theory, this study results indicate that the impact of upper management support on IA's effectiveness is positively and significantly influenced by extrinsic rewards, as demonstrated by H6. Therefore, in addition to providing adequate support to internal auditors, offering appropriate levels of extrinsic rewards can further enhance the influence of upper management support on IA's effectiveness [131]. Moreover, the study found that the link between IAD size and IA's effectiveness (H9) is also positively and significantly moderated by extrinsic rewards as supported by the agency theory. This suggests that a high level of extrinsic rewards in Jordanian public institutions can strengthen the effect of IAD size on the effectiveness of internal auditors.

## 8 Implications

The present study holds several implications for research in the IA area includinding theoretical and practical consequences.

### 8.1 Theoretical implications

The current research elucidates the influence of various empowerment factors—including upper management support, collaboration with external auditors, the size of the Internal Audit Department (IAD), the independence of internal auditors, and extrinsic rewards—on bolstering the effectiveness of internal auditors. In addition, the findings demonstrate that external incentives significantly enhance the proficiency of internal auditors, particularly in circumstances such as Jordan where poverty is pronounced, remuneration is diminished, and costs are high. Although a number of studies have examined the relationship between extrinsic rewards and employee performance across diverse industries, few have examined the importance of extrinsic rewards in enhancing the effectiveness of internal auditors. Additionally, this study reveals that different degrees of extrinsic rewards result in different magnitudes of empowerment impacting internal audit functions. The results of this study demonstrate that different levels of extrinsic rewards modulate the impact of empowerment on internal auditor efficacy.

### 8.2 Practical implications

As an implications of the present study's findings, the stakeholders or decision-makers must consider a sufficient level of such empowerments to achieve high IA's effectiveness. Furthermore, it is imperative for policymakers to give due consideration to the empowerment of internal auditors, ensuring their capacity to render auditing services proficiently. In this vein, the policy-makers should work on providing proper strategies to enhance the independence and create a fair extrinsic rewards system, as well as provide a proper tools and means to facilitate the cooperation between the internal and external parties.

## 9 Limitation and future research recommendations

Nevertheless, like any other researches, there are some limitations to the current investigation. First, the current study focuses on the empowerments that contribute to the effectiveness of IAs, namely, UMS, external auditor collaboration, IAD size, internal auditor independence, and extrinsic compensation. As a result, further research may uncover other empowerments. Second, the current study investigates research hypotheses on the efficacy of internal auditors in terms of completing appointed tasks. To be effective, furthere works might focus on the IA's role in preventing corruption. Third, the present study has addressed the traditional aspects of internal auditors' work. Future research might delve into the electronic dimensions of the internal audit function, especially in the context of the digital advancements of the 21st century. Additionally, this investigation is predicated on the Jordanian public sector as its primary study population.Future research could apply the model of this study in other domains or compare the results between different sectors. In addition, future studiea also could pay attention to one more model for determining the quality of the internal audit, which model can be indicated as an alternative model for testing the quality of the internal audit, namely: the VAIA model. Fifth, future studies could explore the impact of Artificial Intelligence and cloud computing on the profession of internal audit, especially in light of the renaissance that the world is witnessing today. In conclusion, it should be emphasized that the findings of the present study are specific to the internal audit setting within the Jordanian public sector. Therefore, caution should be exercised when applying these results to other contexts. It is likely that the outcomes could vary significantly in different sectors or countries. Notwithstanding these constraints, the current study demonstrates the need of scientifically examining and enabling IAs, as validated by the RBV.

## Data availability statement

Data will be made available on request.

## CRediT authorship contribution statement

**Hamza Alqudah:** Conceptualization, Writing – original draft. **Noor Afza Amran:** Conceptualization, Investigation, Writing – original draft. **Haslinda Hassan:** Conceptualization, Investigation, Writing – original draft. **Abdalwali Lutfi:** Formal analysis, Funding acquisition, Project administration, Writing – review & editing. **Noha Alessa:** Writing – review & editing. **Mahmaod alrawad:** Formal analysis, Validation, Writing – review & editing. **Mohammed Amin Almaiah:** Formal analysis, Validation, Writing – review & editing.

## Declaration of competing interest

The authors declare that they have no known competing financial interests or personal relationships that could have appeared to influence the work reported in this paper.

## Figures and Tables

**Table:** 

Internal Auditors' Effectiveness	The financial managers' responses on the IA effectiveness's aspects adapted from Alzeban and Gwilliam [20], includes the development of the organization's productivity, the capability to plan, assessment the compatibility between the results and the assigned objectives, valuation and improvement of risk management, a response to the IA recommendations, assessment of IC systems, and recommendations for improvements.
Upper management support	Six items measured this construct adapted from Alzeban and Gwilliam [20], including (1) upper management is involved in the plan of the IA, (2) Upper management helps the internal auditors perform their responsibilities, (3) The reply to IA's reports by the upper management is routine. (4) Internal auditors give upper management adequate and appropriate reports regarding the work they do and the recommendations made, (5) IAD has an adequate budget to actively carry out its duties, and (6) IAD size is sufficient to execute its responsibilities effectively.
External audit cooperation	Nine items measured this construct adapted from Alzeban and Gwilliam [20], including (1) External auditors have a favorable attitude towards IA, (2) External auditors are supportive and friendly, (3) External and internal auditors discuss the timing of work in which they have a shared interest, (4) External auditors are ready to provide internal auditors a chance to demonstrate their concerns, (5) External auditors trust the IA reports, (6) External auditors review their plans with the internal auditors, (7) External and internal auditors share their working papers, (8) External and internal auditors meet regularly, and (9) Senior management assists in improving the internal-external auditors' cooperation.
Independency	Nine items measured this variable adapted from Alzeban and Gwilliam [20], including (1) IA staffs are independent of accomplishing their professional duties and obligations, (2) The head of IAD reports to a level within the organization that allows the IA achieve its responsibilities, (3) The CAE has a direct connection with the Minster (in ministries) and the General Manager (in the government departments), (4) The IAD has a direct connection with the upper management besides the finance director, (5) Internal auditors seldom face intervention by the management while they execute their work, (6) Conflict of interest is seldom happening in the internal auditors' work, (7) IA staff have unrestricted access to all employees and departments in the organization, (8) The board of directors accepts the appointment and replacement of the CAE, and (9) Internal auditors are not asked to do non-audit functions.
The IAD's size	One item measured this variable adopted from Alzeban and Gwilliam [20] by the number of IA staff worked within each IAD.
Extrinsic rewards	Eight items measured this construct adapted from Malhotra et al. [99], including (1) The working conditions are sufficient to execute a good internal auditor's job, (2) Internal auditors are happy with the working conditions at their workplace, (3) Internal auditors are pleased with the amount of pay they get for the job they do, (4) Internal auditors are satisfied with their pay compared with other organizations they know, (5) Internal auditors feel they are paid fairly compared with the work they do, (6) Internal auditors are pleased with the fringe benefits package, (7) The fringe benefits package is as good as other organizations offer, (8) Internal auditors feel that the promotion policy is good, and (9) There is an enough opportunity for advancement on the internal auditors' job

**figs1:**
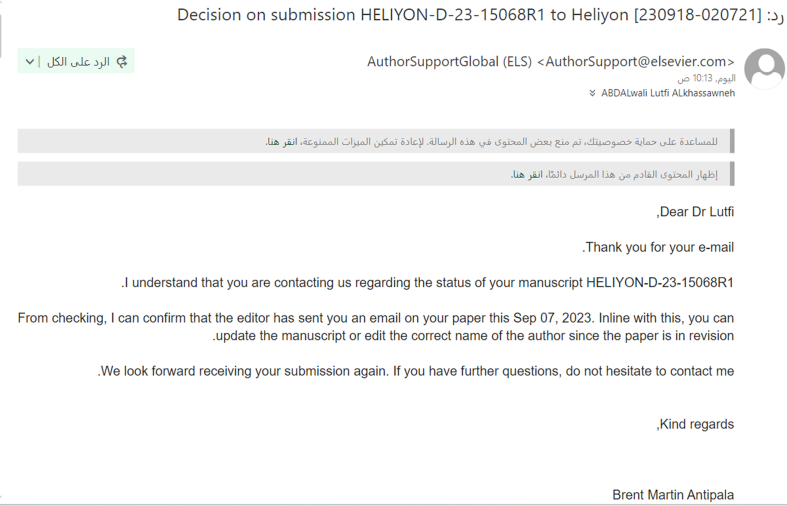


## Footnotes

Appendix A Supplementary data to this article can be found online at https://doi.org/10.1016/j.heliyon.2023.e20497.
