# Supplementary material for: Examining the critical factors of internal audit effectiveness from internal auditors’ perspective: Moderating role of extrinsic rewards
Source: Heliyon. 2023 Sep 30;9(10):e20497. doi: 10.1016/j.heliyon.2023.e20497 (PMC10570590; doi:10.1016/j.heliyon.2023.e20497)
Supplement: Multimedia component 1 [file mmc1.pdf]

Questionnaire to IA's Managers

Code	Scale Items<br>Internal Audit Effectiveness	Strongly disagree				Strongly agree
IAE1	Internal audit improves organizational performance.	1	2	3	4	5
IAE2	Internal audit reviews operations and programmers to ascertain whether results are consistent with established objectives and goals.	1	2	3	4	5
IAE3	Internal audit determines the adequacy and effectiveness of the government institution's systems of internal accounting and operating controls.	1	2	3	4	5
IAE4	Internal audit reviews the accuracy and reliability of financial reports.	1	2	3	4	5
IAE5	Internal audit reviews the compliance with policies, plans, procedures and regulations.	1	2	3	4	5
IAE6	Internal audit reviews the compliance with applicable external laws, and regulations.	1	2	3	4	5
IAE7	Internal audit reviews the means of safeguarding assets.	1	2	3	4	5
IAE8	Internal audit evaluates and improves the effectiveness of risk management.	1	2	3	4	5
IAE9	Internal audit reviews the economical, effective and efficient use of resources.	1	2	3	4	5
IAE10	Internal audit evaluates the internal control system.	1	2	3	4	5
IAE11	Internal audit makes recommendations for improving the internal control system when appropriate.	1	2	3	4	5
IAE12	Internal audit improves the government institution's productivity.	1	2	3	4	5
IAE13	Internal audit develops appropriate annual audit plans.	1	2	3	4	5
IAE14	Timely action is taken to implement the recommendations of the internal audit report.	1	2	3	4	5
IAE15	Internal audit provides adequate follow-up to ensure that appropriate corrective action is taken and that it is effective.	1	2	3	4	5

Code	Scale Items<br>Top Management Support	Strongly disagree				Strongly agree
TMS1	Top management supports internal audit to perform its duties and responsibilities.	1	2	3	4	5
TMS2	Top management is involved in the internal audit plan.	1	2	3	4	5
TMS3	Internal audit provides senior management with sufficient, reliable and relevant reports about the work they perform and recommendations made.	1	2	3	4	5
TMS4	The response to internal audit reports by the senior management is reasonable.	1	2	3	4	5
TMS5	Internal audit department is large enough to successfully carry out its duties and responsibilities.	1	2	3	4	5
TMS6	Internal audit department has sufficient budget to successfully carry out its duties and responsibilities.	1	2	3	4	5

Code	Internal Auditors' Independence	1	2	3	4	5
IAI1	Internal audit staffs are sufficiently independent to perform their professional obligations and duties.	1	2	3	4	5
IAI2	The head of internal audit reports to a level within the organization that allows the internal audit to fulfill its responsibilities.	1	2	3	4	5

**IAI3**	The head of internal audit has direct contact to the Minster (in ministries), to the President (in universities) and to the General manager (in government departments).	1	2	3	4	5
**IAI4**	The internal audit department has direct contact with senior management other than the finance director.	1	2	3	4	5
**IAI5**	Conflict of interest is rarely present in the work of internal auditors.	1	2	3	4	5
**IAI6**	Internal auditors rarely face interference by management while they conduct their work.	1	2	3	4	5
**IAI7**	Internal audit staffs have free access to all departments and employees in the organization.	1	2	3	4	5
**IAI8**	The board of directors (the President in Government Organizations) approves the appointment and replacement of the head of internal auditing.	1	2	3	4	5
**IAI9**	Internal auditors are not requested to perform non-audit functions.	1	2	3	4	5

**Code**	**Internal-External Auditors' Cooperation**	**1**	**2**	**3**	**4**	**5**
**IEAC1**	External auditors are friendly and supportive.	1	2	3	4	5
**IEAC2**	External auditors have a good attitude towards internal auditors.	1	2	3	4	5
**IEAC3**	External auditors are willing to give internal auditors an opportunity to explain their concerns.	1	2	3	4	5
**IEAC4**	External auditors and internal auditors consult on the timing of work in which they have a mutual interest.	1	2	3	4	5
**IEAC5**	External auditors discuss their plans with IA.	1	2	3	4	5
**IEAC6**	External auditors rely on IA work and reports.	1	2	3	4	5
**IEAC7**	External auditors and internal auditors meet on a regular basis.	1	2	3	4	5
**IEAC8**	External auditors and internal auditors share their working papers.	1	2	3	4	5
**IEAC9**	Senior management helps to promote effective co-operation between internal audit and External auditors.	1	2	3	4	5

**Code**	**Extrinsic Reward**	**1**	**2**	**3**	**4**	**5**
**ER1**	The working conditions are adequate to perform a good internal auditor's job.	1	2	3	4	5
**ER2**	Internal auditors are satisfied with the working conditions at their workplace.	1	2	3	4	5
**ER3**	Internal auditors are satisfied with the amount of pay they receive for the job they do.	1	2	3	4	5
**ER4**	Internal auditors are satisfied with their pay considering other organizations they know of.	1	2	3	4	5
**ER5**	Internal auditors feel they are paid fairly considering the work they do.	1	2	3	4	5
**ER6**	Internal auditors are satisfied with the fringe benefits package.	1	2	3	4	5
**ER7**	The fringe benefits package is as good as other organizations offer.	1	2	3	4	5
**ER8**	Internal auditors feel that the promotion policy is good.	1	2	3	4	5

**ER9**	There is enough opportunity for advancement on internal auditors' job	1	2	3	4	5

Code	Task Complexity	1	2	3	4	5
**TC1**	Internal auditors frequently deal with unstructured audit operations.	1	2	3	4	5
**TC2**	Internal auditors frequently deal with ad hoc, non-routine audit works.	1	2	3	4	5
**TC3**	The audit operations that internal auditors deal with frequently involve more than one place or branch.	1	2	3	4	5
**TC4**	The audit operations that internal auditors deal with involve carrying out tasks that have never existed in that way before.	1	2	3	4	5
**TC5**	In internal auditors' job, there is a great deal of variety of the problems, issues, or operations for which they need data.	1	2	3	4	5
**TC6**	The audit operations that internal auditors deal with frequently involve more than one department function.	1	2	3	4	5
**TC7**	In internal auditors' work, they frequently have to think about audit issues and the associated data in new ways.	1	2	3	4	5
**TC8**	It is necessary for internal auditors to spend time thinking about how best to address an audit works before they begin an auditing.	1	2	3	4	5

Part B: Demographic and Background Information

Information: Please tick (√)/or fill as appropriate in the provided spaces your exact assessment of the following demographic and background information:

A. Gender: □ Male □ Female

B. Age: □ 20-29 □ 30-39 □ 40-49 □ More than 50 years

C. Education Qualifications

-Education level: □ High school/below □ Bachelor's degree □Master □ PhD

-Specialization: □ Accounting □ Law □ Business □ Management □ Other (Please specify)……………………….

D. Work experience:

□ 1-5 years □ 6-10 years □ 11-15 years □ More than 16 years

E. Job title:

□ Administrative assistant □ Budget specialist □ Technique audit Manager

□ Managerial audit manager □ financial auditor manager □ Head of IA department

□ Others (Please specify)………………………

F. Professional qualification:

□ Certified Internal Auditor (CIA)

□ Certified Public Accountant (CPA)

□ Jordan Certified Public Accountant (JCPA)

□ Certified Financial Examiner (CFE)

□ Certified Management Accountant (CMA)

□ Certified Information System Auditor (CISA) □ Others (Please specify)…………

G. Annual training hours:

□ No training □ <20 □ 21–40 □ 41–80 □ 81–120 □ More than 120

H. Internal audit courses:

□ None □ One course □ Two courses □ Three courses

□ Four or more courses

Please indicate the number of internal auditors in your department:

□ 1–4 □ 5–8 □ 9–12 □ 13–16 □ 17-20 □ More than 20
